# Ulceration of Frænum Linguæ in Pertussis

**Published:** 1879-01

**Authors:** 


Ulceration of Frænum Linguæ in Pertussis.—Dr. W. N. Moccall calls attenticn to this ulceration as a valuable aid to the diagnosis of pertussis. It is not a constant lesion, but exists in about one-half of the cases. Its cause seems to be mechanical; in the cough the frænum is stretched and the tongue is rubbed in a sawing manner on the lower incisors. In any other situation than the front of the frænum it seems to be due to irregular teeth. Bouchut's dictum is "that a child which coughs, and this ulceration on or near the frænum linguæ certainly has whooping-cough."

Dr. C. Elliott has found this ulcer present in only twenty-five per cent. of his cases. He thinks its origin is both follicular and mechanical, but it has been found in children which had not yet cut their teeth.—*Maryland Medical Journal.*

